# Spatiotemporal Characterization of a Fibrin Clot Using Quantitative Phase Imaging

**DOI:** 10.1371/journal.pone.0111381

**Published:** 2014-11-11

**Authors:** Rajshekhar Gannavarpu, Basanta Bhaduri, Krishnarao Tangella, Gabriel Popescu

**Affiliations:** 1 Quantitative Light Imaging Laboratory, Department of Electrical and Computer Engineering, Beckman Institute for Advanced Science and Technology, University of Illinois at Urbana-Champaign, Urbana, Illinois, United States of America; 2 Department of Pathology, Christie Clinic, and University of Illinois at Urbana-Champaign, Urbana, Illinois, United States of America; University of Pécs Medical School, Hungary

## Abstract

Studying the dynamics of fibrin clot formation and its morphology is an important problem in biology and has significant impact for several scientific and clinical applications. We present a label-free technique based on quantitative phase imaging to address this problem. Using quantitative phase information, we characterized fibrin polymerization in real-time and present a mathematical model describing the transition from liquid to gel state. By exploiting the inherent optical sectioning capability of our instrument, we measured the three-dimensional structure of the fibrin clot. From this data, we evaluated the fractal nature of the fibrin network and extracted the fractal dimension. Our non-invasive and speckle-free approach analyzes the clotting process without the need for external contrast agents.

## Introduction

Fibrin is a biopolymer that constitutes the structural basis of the hemostatic plug, which prevents excessive blood loss upon vascular injury [1], [2]. During blood coagulation, the protein *fibrinogen* in blood plasma is converted to *fibrin* by the action of thrombin [3], [4]. As a result, a three-dimensional network or *gel* is formed, which comprises of entangled, branching fibrin fibers and binds the platelets to stop bleeding. In addition, this mesh anchors the neutrophils, macrophages, and fibroblasts to remove dead tissues and infectious agents during wound repair [4], [5]. The fibrin structure has been associated with vascular disease [6], stroke [7], diabetes [8], and thromboembolic disorders [9], [10]. In addition to clinical significance for hemostasis, fibrin can be used as scaffolding material for tissue engineering [11], [12], for drug delivery [13], [14], and in tissue patterning [15], [16]. Thus, studying the dynamics of fibrin clot formation and its associated morphology is of great basic science and clinical significance.

The prominent techniques for analyzing fibrin network include scanning electron microscopy [17], atomic force microscopy [18], [19], x-ray scattering [20], [21], and neutron scattering [22], [23]. Although these techniques provide high resolution detail, they involve bulky experimental configurations, require dedicated infrastructure, have low throughputs, and exhibit limited applicability to three-dimensional (3D) visualization of the fibrin structure. Alternatively, optical approaches have been proposed. Light scattering [24], [25] is an important optical technique, which has been used for studying fibrin networks. Similarly, speckle characteristics of the scattered light have also been investigated for fibrin studies [26]. Due to limited optical sectioning provided by coherent illumination, the main limitation of these approaches is that they are not suitable for direct three-dimensional imaging of the fibrin network. Another class of optical techniques based on confocal fluorescence microscopy [27]–[30], deconvolution fluorescence microscopy [31], and total internal reflection fluorescence microscopy [32] have been proposed for studying the 3D structure and dynamics of fibrin polymerization. However, they rely on the addition of exogenous labeling agents to the specimen, which is subject to photobleaching and phototoxicity. Recently, differential interference contrast microscopy methods [33], [34] and flow assay techniques [35] were proposed for label-free fibrin studies.

Here, we present a novel approach for assessing the spatiotemporal characteristics of a fibrin clot. Our approach relies on quantitative phase imaging (QPI), where we retrieve the fibrin clot structure via an interferometric measurement. Importantly, the nanoscale optical path length changes introduced by the clot result in observable phase shifts of the illumination field [36]. The imaging modality in our approach is based on spatial light interference microscopy (SLIM) [37], [38], which has been applied previously for biological studies including red blood cell morphology [39], cell growth monitoring [40]–[42], cellular tomography [43] etc. Using QPI, we monitored the formation of a fibrin clot in real-time and analyzed the growth characteristics using the spatial power spectrum. We also measured the three-dimensional structure of the clot by capturing depth-resolved quantitative phase images and studied the associated fractal properties.

## Methods

### Ethics Statement

The studies have been performed in accordance with the procedure approved by the Institutional Review Board at University of Illinois at Urbana-Champaign (IRB Protocol Number: 10571). The blood sample used in this research project was procured after securing a signed general consent from the donor, which allows the specimen to be used for educational and research purposes.

### Sample preparation

Human venous blood sample was collected in a trisodium citrate tube. The citrate acts an an anti-coagulant to prevent clotting before imaging. The whole blood was initially centrifuged at 1500×g for about 5 minutes. Subsequently, we aspirated the top layered plasma, collected it in another microcentrifuge tube, and performed centrifugation for another 5 minutes at 1500×g. The double centrifugation was done to minimize the residual red blood cells and platelets. From the spun tube, the top layered plasma was collected for the experiment. A sample chamber was created by punching a hole in a double sided scotch tape and sticking one side of the tape onto a cover slip. Subsequently, we pipetted 3 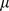
l of plasma into the chamber and added 0.5 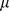
l of 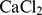 (0.025 M, 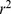 Diagnostics). Blood clotting is initiated by the addition of calcium, and is dependent upon the activation of FXII by glass (contact activation) [44], [45]. Then, we sealed the top of the chamber using another cover slip to reduce evaporation, and transferred the sample to the SLIM setup for imaging.

### Imaging and Image Processing

**Figure 1:**
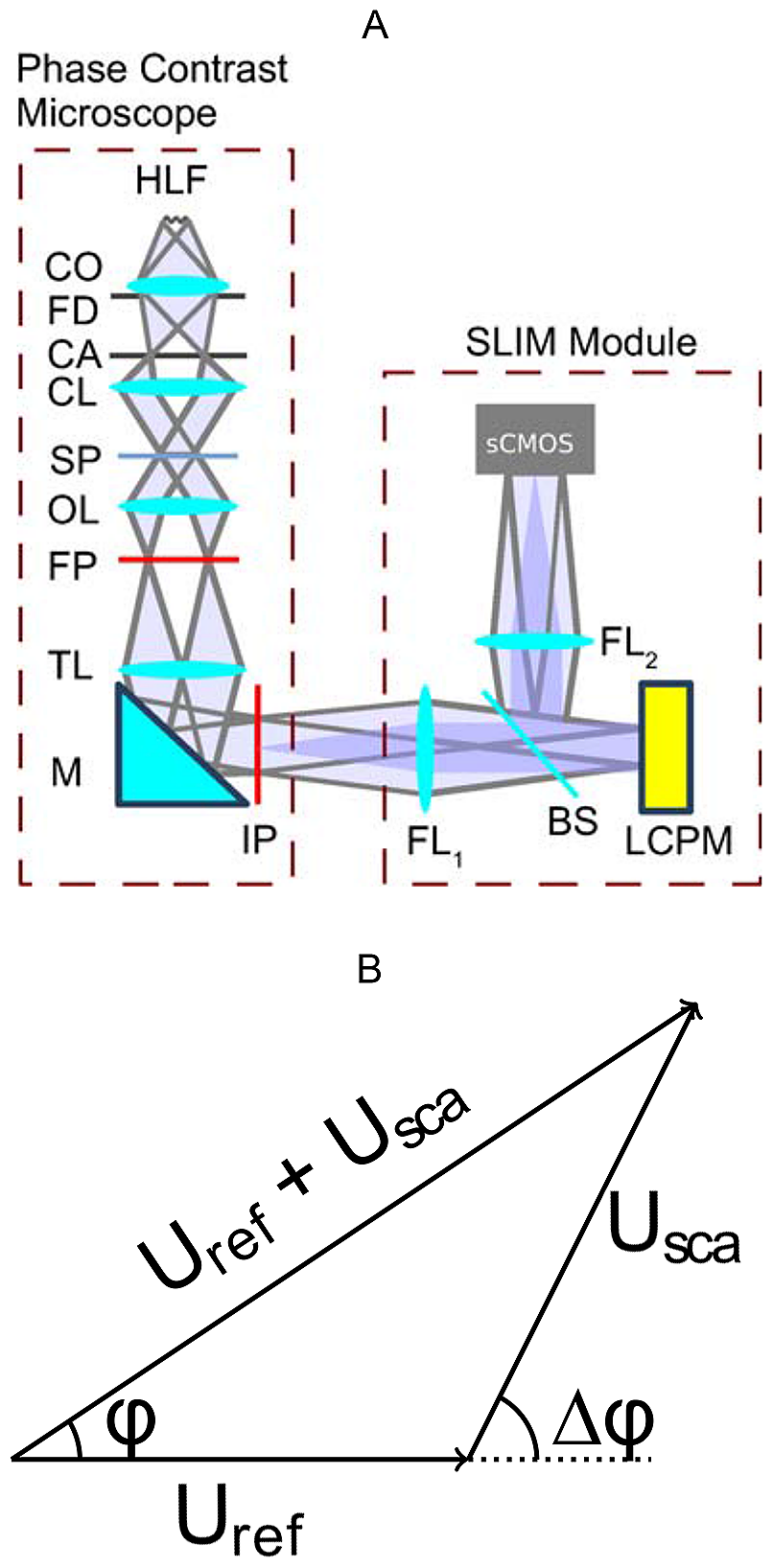
SLIM system. (A) Imaging setup. HLF: Halogen Lamp Filament, CO: Collector lens, FD: Field Diaphragm, CA: Condenser Annulus, CL: Condenser Lens, SP: Specimen, OL: Objective Lens, FP: Back Focal Plane of Objective, TL: Tube Lens, M: Mirror, IP: Image Plane, FL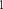: Fourier Lens 1, FL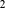: Fourier Lens 2, BS: Beam Splitter, LCPM: Liquid Crystal Phase Modulator. (B) Coherent superposition of scattered and unscattered waves.

The imaging setup is shown in Fig. 1(A). Here, SLIM works as an add-on module to a commercial phase-contrast microscope (Zeiss Axio Observer Z1) with a 40X microscope objective (Zeiss, Ph 2, NA = 0.75). It relies on the spatial decomposition of the image field into its scattered and unscattered components. In addition to the conventional 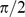 shift introduced in phase contrast microscopy, three phase shifts in increments of 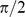 were introduced. The additional phase modulation was achieved by using a reflective liquid crystal phase modulator (LCPM, Boulder Nonlinear Systems). The LCPM is placed in the Fourier plane of the SLIM module. This plane is conjugate to the back focal plane of the microscope objective which contains the phase contrast ring. For effective control of the additional phase delay between the scattered and unscattered components, the active pattern on the LCPM is computed to precisely match the size and position of the phase contrast ring image. The 4f system formed by lens FL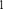 (focal length 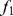 = 150 mm) and lens FL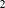 (focal length 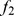 = 200 mm) provides additional magnification of 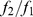.

We used a scientific-grade complementary metal oxide semiconductor (sCMOS) camera (Andor Zyla) for capturing images. The camera records the superposition of the scattered field 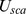 and unscattered field 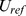, as shown by the vectorial representation in Fig. 1(B). In the figure, 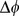 is the phase difference between 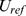 and 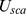, whereas 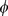 is the phase of the image field, the quantity of interest in QPI. The four 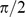 phase-shifted images can be represented as,
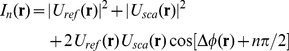
(1)where 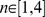. We can compute 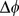 as,
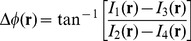
(2)


Denoting 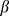 as the ratio of the amplitudes of the scattered and unscattered fields, i.e. 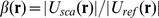, the phase of the image field can be obtained as,
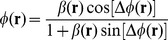
(3)


The measured phase is dependent on the refractive index and thickness of the sample as
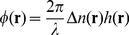
(4)where 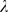 is the mean wavelength, 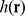 is the thickness and 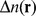 is the refractive index difference between the sample and medium. As phase measurement is influenced by refractive index, it is important to minimize the transparent blood cells in the sample to clearly map the fibrin structure. More details about SLIM construction and working are reported elsewhere [38].

### Power spectrum and fractal analysis

For analyzing the temporal dynamics of the fibrin clot, the 2D spatial power spectrum as a function of time was computed using the surface integral,
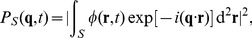
(5)where 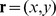 and 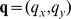, with 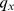 and 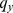 being angular spatial frequencies. Further, the spatial power spectrum was normalized by dividing the spectrum data by the maximum value. Subsequently, the radial average 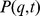 is obtained by averaging the normalized 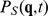 along 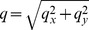.

For analyzing the three-dimensional structure of the fibrin network, the 3D spatial power spectrum was computed using the volume integral,
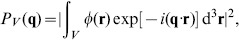
(6)where 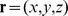 and 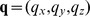. Subsequently, the spherical average 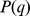 was ascertained by averaging the normalized 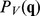 along 
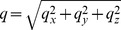
.

For fractal analysis of the 3D fibrin network, the spherically averaged spatial power spectrum 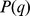 vs 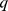 was plotted on a log-log scale. The presence of a linear behavior on log-log scale, or equivalently a power law decay of the form 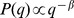 is suggestive of the underlying fractal nature of the 3D network [46]. The decay exponent 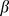 was computed using a linear regression fit on the logarithmic scale. To quantify fractal characteristics, the fractal dimension 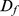 was obtained from the decay exponent using 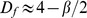 [46]. Fractal dimension can be considered to be a measure of compactness of the network in three-dimensional space.

All image processing tasks and mathematical operations were performed in MATLAB. The regression calculations were performed using MATLAB's built-in optimization routines.

## Results

**Figure 2:**
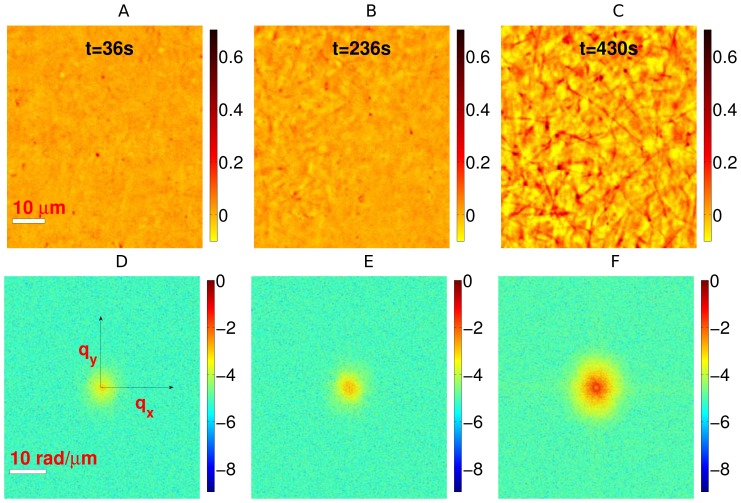
Fibrin clot formation. (A–C) illustrate the measured phase in radians at different time instants. (D–F) show the corresponding normalized log power spectra. On the top row, we see the evolution of a clot in time. This is accompanied by power spectrum broadening, shown on the bottom row.

After initiating the clotting process, as discussed above, we obtained time-lapsed quantitative phase images at 1.2 Hz, each with a field of view (FOV) of approximately 60 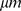 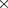 60 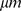. The quantitative phase maps at three different time instants are shown in Fig. 2. During the initial period of clotting, as shown in Fig. 2A, there is no significant spatial variation in phase. As clotting progresses, we observe that the fluctuations in refractive index, induced by fibrin polymerization, manifest as corresponding changes in the measured phase. This is evident from the phase maps at later time instants (Figs. 2B and C) where the emergence of a mesh structure is clearly visible. The growth of fibrin clot is also shown in Movie S1. Note that for dynamics studies, an important requirement is the temporal stability of the measuring system. SLIM offers such capability due to its robustness against temporal noise granted by the common-path geometry [37]. In addition, SLIM significantly reduces the speckle noise because of the broadband illumination. These features enable the assessment of dynamics of fibrin clot formation with high sensitivity.

**Figure 3:**
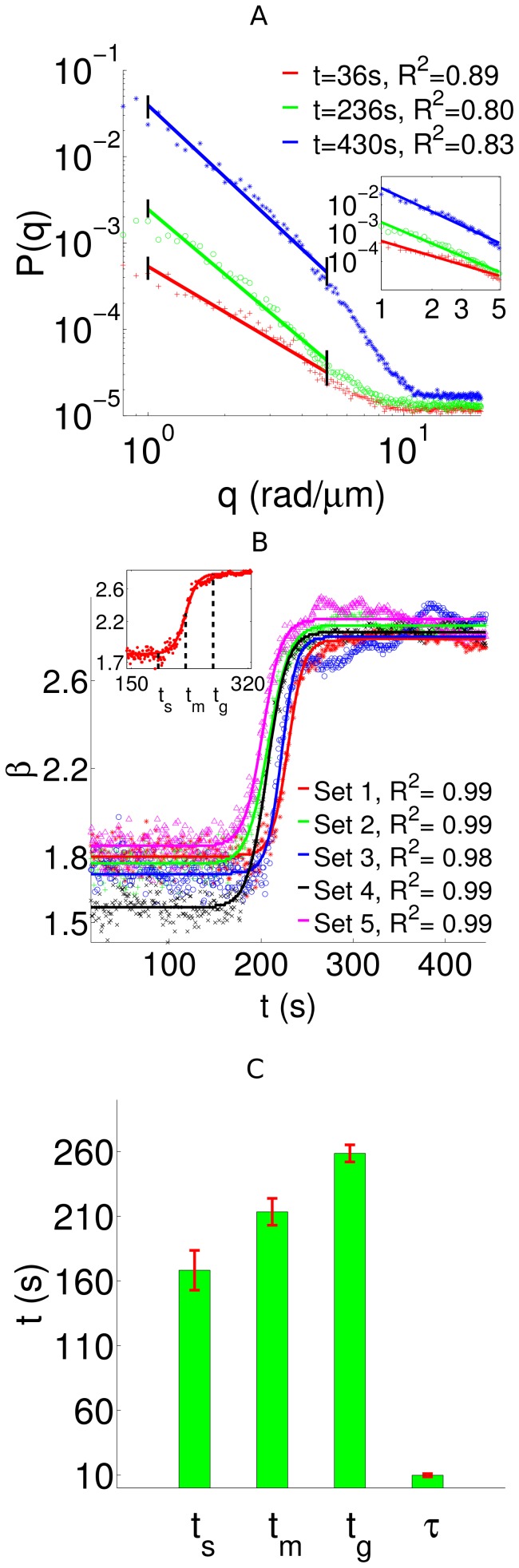
Fibrin temporal characteristics. (A) The radially averaged power spectra for different time instants on a log-log scale. For low 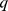, these spectra exhibit a power law behavior, 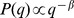, as confirmed by the linear fits (solid lines) on the log-log scale. This power law decay regime for the three time instants is shown in the inset. (B) The decay exponent 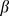 as a function of time 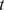 for five different datasets. The solid lines indicate sigmoidal fits. The inset shows a temporal interval of one dataset, where the two-stage behavior of fibrin polymerization is clearly evident. (C) The measured values of 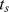, 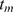, 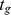 and 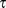. The errorbars indicate the standard deviation.

To analyze the temporal evolution of the fibrin network, we used the spatial power spectrum of the quantitative phase map. The normalized power spectra (log scale) for the three quantitative phase maps, shown in Figs. 2A–C, are presented in Figs. 2D–F respectively. From these figures, we observe a distinct broadening of the power spectrum, as the clot forms. This can be attributed to the fact that the formation of an interconnected fibrin mesh is accompanied by an increased spectral contribution to higher spatial frequencies. Hence, power spectrum of the quantitative phase carries important information about the growth characteristics of the fibrin network. This is clearly illustrated by the radially averaged power spectrum 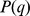, as shown in Fig. 3A. Two important observations can be made from the plot: (1) for low 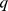, the power spectrum varies linearly with 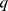 on log-log scale, which indicates a power law decay of the form 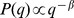; (2) the decay exponent 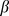 varies with time as the fibrin polymerizes. Here, we are mainly interested in the low 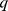 region, as it corresponds to the large length scales at which fibrin fibers cross-link.

To quantify fibrin polymerization, we obtained time-lapsed quantitative phase images in different regions and computed the power spectrum decay exponent 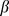 as a function of time for each of these datasets. The temporal evolution of 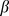 is shown in Fig. 3B. Remarkably, the temporal response for each dataset shows a sigmoid shape, characterized by distinct lower and upper saturation values, along with an intermediate transition period. These observations reveal a two-stage dynamic behavior for fibrin polymerization, indicating the liquid to gel transition. Subsequently, we applied a sigmoidal fit for 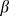 as,
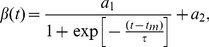
(7)where 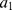 is the response range which decides the upper plateau, 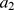 indicates the lower plateau of the sigmoid, 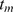 is the time instant corresponding to the steepest growth rate, and 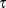 is the time constant of the process, which controls the transition period from one stage to another. The sigmoidal fits, obtained using non-linear regression, are also shown in the figure. In particular, the figure inset illustrates the computed 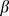 and its fit for a dataset, where three temporal regimes are clearly visible, as follows. (1) The initial period of clotting characterized by 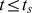, when 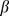 has a lower plateau indicating minimal clot formation. (2) The intermediate period 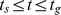 when 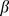 rises rapidly, which signifies the monomer (liquid) to polymer (gel) transition. (3) The saturation period 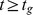 when 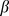 reaches an upper plateau, indicating that the gel structure has formed. Here, 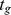 can be considered as an indicator of the *clotting* time [47]. To evaluate 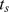 and 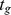, we considered the time instants at which 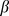 is below or above the upper and lower saturation values by 1% of the response range (see Text S1). The different temporal parameters characterizing the dynamics of fibrin polymerization are summarized in Fig. 3C. From these results, we can infer that QPI provides interesting insights into the dynamics of fibrin network formation. Interestingly, the observed two-level system is consistent with previously reported clotting behavior [47]–[49], where sigmoid growth characteristics were observed, and the clotting parameters were shown to be physiologically relevant [48].

**Figure 4:**
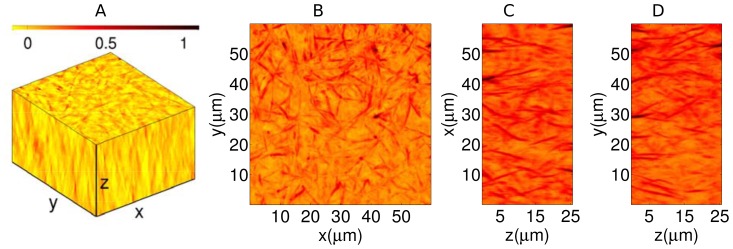
Three-dimensional clot imaging. (A) The 3D structure of fibrin network. The corresponding maximum value projections on the 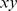, 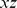, and 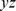 planes are shown in (B), (C), and (D) respectively. The nodal points of the network are clearly visible. The colorbar indicates quantitative phase values in radians.

Next, we studied the three-dimensional structure of the fibrin network. We obtained a z-stack of quantitative phase images by scanning a fully formed fibrin clot along the axial (depth) direction in steps of 0.1 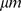 at 6.6 Hz. As demonstrated recently, SLIM offers excellent optical sectioning due to broadband light, resulting in reliable tomographic reconstruction of transparent specimens [43]. We acquired a single 3D image (FOV approx. 60 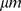 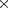 60 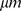 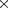 25 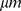) in 38 seconds, which underscores the high throughput of the system. By repeating the above procedure, we obtained ten different 3D phase maps of fibrin networks. Figure 4A illustrates the three-dimensional structure of one such fibrin network. The branching fibrin fibers and the inter-connected nodes are clearly visible in the figure. For better illustration, the depth-scanned quantitative phase images are also shown in Movie S2. The maximum value projections of the 3D quantitative phase image on the 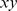, 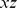 and 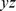 planes are shown in Figs. 4B–D respectively. Since the nodes indicate the cross-linking of several fibrin fibers, resulting in increased local density, these regions are characterized by large phase values. Hence, the maximum value projection maps shown here highlight the nodal points of the network along the three dimensions and provide topological information about the fibrin gel.

**Figure 5:**
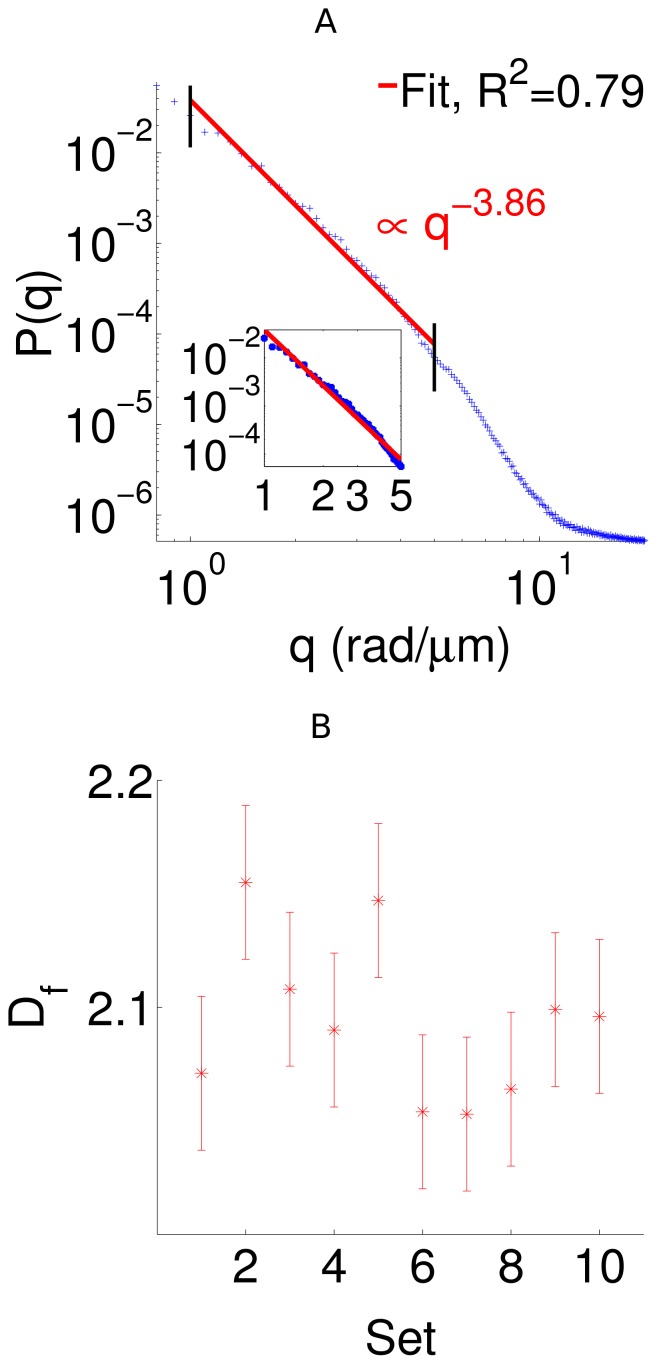
3D structural characteristics of fibrin network. (A) Spherically averaged normalized 3D power spectrum for a dataset on the log-log scale. The inset shows the linear regime of the power spectrum indicating the fractal nature. The solid line represents the linear fit. (B) The computed fractal dimensions for different data sets. The errorbars indicate the standard deviation.

Using the 3D QPI data, we obtained the spherically averaged spatial power spectrum for the different datasets. In Fig. 5A, we show the power spectrum plot for a particular dataset. The power spectrum plots for the other nine datasets are shown in Fig. S2. For all these data, at large length scales, we observed a power law decay behavior, which indicates the fractal nature of the fibrin clot. It is interesting to note that the fractal characteristics of the fibrin network have also been previously observed using neutron scattering [22] and clot rheology [50]. Using our technique, the measured values of the fractal dimension for the ten different sets are shown in Fig. 5B, resulting in 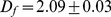 (mean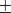 standard deviation). The fractal dimension 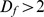 is consistent with a surface fractal structure and indicates that the fibrin fibers exhibit rough surfaces at the observed length scales. Note that the fractal studies of the fibrin clot are important since fractal characteristics have been suggested as potential biomarkers for clotting related disorders [50].

## Summary and Discussions

We demonstrated a novel approach based on quantitative phase imaging to study the fibrin network in a non-invasive and label-free manner. Using QPI and power spectrum analysis, we quantified the temporal evolution of a fibrin clot and presented a simple mathematical description of the two-stage growth characteristics of fibrin polymerization. The high throughput of our measurement system allows real-time monitoring of fibrin networks over large area. In addition, our system provides high temporal sensitivity for dynamics studies. Furthermore, we investigated the 3D structure of fibrin network and studied the associated fractal properties. For such studies, our technique provides full 3D imaging capability with good spatial sensitivity and non-requirement of exogenous markers.

For the current studies, we used blood plasma as the sample, which is mostly transparent, and free from residual blood cells. Even if stray platelets are present, their adverse effect on the accuracy of the spatio-temporal analysis would not be high. This is because of the much smaller platelet dimensions in contrast to the size of the branching fibrin network. Also, the effect of spherical averaging of the spatial power spectrum would mitigate the influence of phase variations induced by the platelets. Further, one can measure whole blood with our technique, provided an area devoid of cells is imaged, such as on a smear. In addition, slight dilution of the blood would be helpful to provide entire field of view without cells.

The QPI methodology opens up interesting possibilities for future research regarding fibrin clots. The fibrin microstructure could be explored by altering the concentrations of fibrinogen and calcium ions, and studying the corresponding effect on clot attributes such as the degree of crosslinking and lateral association, pore size etc. In addition, since our technique provides a full-field quantitative phase image without the need of pixel by pixel scanning, the spatial power spectrum analysis can be performed locally, by selecting a region of interest, instead of the whole image. This could provide important region-specific information such as the inhomogeneities within a clot structure. Another future research area is to investigate the temporal parameters associated with clot formation for pathological relevance by comparing healthy and unhealthy specimens.

To summarize, quantitative phase imaging offers several advantages for measuring clot characteristics, and exhibits great potential for fibrin related biological and clinical applications.

## Supporting Information

Figure S1
**Sigmoidal model of the decay exponent** 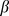 **as a function of time.**
(EPS)

Figure S2 **Spherically averaged normalized 3D power spectrum on a log-log scale for nine datasets in (A-I).** The respective insets show the power law decay regions (linear regime on log-log scale) indicating the fractal nature. The solid lines represent linear fits.
(EPS)

Text S1 **Description of sigmoid parmeters.**
(PDF)

Movie S1 **Temporal growth of a fibrin clot is monitored using quantitative phase imaging.** The formation of a cross-linked fibrin mesh with time can be clearly observed. The colorbar indicates quantitative phase in radians.
(AVI)

Movie S2 **Depth-scanned quantitative phase images of the fibrin clot.** The 3D structure becomes evident as z is varied. The colorbar indicates quantitative phase in radians.
(AVI)
