# Supplementary material for: Spatiotemporal Characterization of a Fibrin Clot Using Quantitative Phase Imaging
Source: PLoS One. 2014 Nov 11;9(11):e111381. doi: 10.1371/journal.pone.0111381 (PMC4227684; doi:10.1371/journal.pone.0111381)
Supplement: Text S1 — Description of sigmoid parmeters. (PDF) [file pone.0111381.s003.pdf]

Description of sigmoid parameters

To quantify the growth characteristics of the fibrin network, the power spectrum decay exponent, i.e. β, was modeled as a sigmoidal function,

$$\beta(t) = \frac{a_1}{1 + \exp\left[-\frac{(t - t_m)}{\tau}\right]} + a_2 \tag{1}$$

This growth curve is shown in Fig. S1. It is evident that the sigmoid function is bounded by the asymptotic lines $\beta = a_2$ (lower plateau) and $\beta = a_1 + a_2$ (upper plateau), and a_1 represents the response range. The time instant t_m indicates the inflection point of the sigmoid, where the first derivative $d\beta/dt$ attains its peak, and hence corresponds to the maximum gain. Further, t_s and t_g represent the lower and upper temporal bounds of the sigmoidal growth such that there is no appreciable increase or decrease in β for $t > t_g$ or $t < t_s$. To gain a better insight about these bounds, we define t_s and t_g as the time instants when β lies above or below the lower and upper plateaus by a finite fraction, say γ, of the response range. For the lower temporal bound, we have

$$\beta(t_s) = a_2 + \gamma a_1 \tag{2}$$

or equivalently,

$$\gamma = \frac{1}{1 + \exp\left[-\frac{(t_s - t_m)}{\tau}\right]} \tag{3}$$

which evaluates to,

$$t_s = t_m - \tau \log\left[\frac{1-\gamma}{\gamma}\right] \tag{4}$$

Similarly, for the upper bound, we start with,

$$\beta(t_g) = a_1 + a_2 - \gamma a_1 \tag{5}$$

implying,

$$1 - \gamma = \frac{1}{1 + \exp\left[-\frac{(t_g - t_m)}{\tau}\right]} \tag{6}$$

which yields,

$$t_g = t_m + \tau \log\left[\frac{1-\gamma}{\gamma}\right] \tag{7}$$

From Eq.(4) and Eq.(7), the temporal bounds of the sigmoid function can be determined. For analyzing the temporal characteristics of blood coagulation, we used $\gamma = 0.01$, which provides the bounds as $t_m \pm 4.59\tau$. Interestingly, the dynamics of blood coagulation is mostly dominant in the temporal interval $t \in [t_s, t_g]$, whose width is decided by the time constant τ, and where the steepest change occurs around $t = t_m$.
